# Promotion of chloroplast proliferation upon enhanced post-mitotic cell expansion in leaves

**DOI:** 10.1186/1471-2229-13-143

**Published:** 2013-09-28

**Authors:** Kensuke Kawade, Gorou Horiguchi, Naoko Ishikawa, Masami Yokota Hirai, Hirokazu Tsukaya

**Affiliations:** 1 Department of Biological Sciences, Graduate School of Science, University of Tokyo, 7-3-1 Hongo, Bunkyo-ku, Tokyo 113-0033, Japan; 2 Department of Life Science, College of Science, Rikkyo University, 3-34-1 Nishi-Ikebukuro, Toshima-ku, Tokyo 171-8501, Japan; 3 Research Center for Life Science, Rikkyo University, 3-34-1 Nishi-Ikebukuro, Toshima-ku, Tokyo 171-8501, Japan; 4 RIKEN Center for Sustainable Resource Science, 1-7-22 Suehiro-cho, Tsurumi-ku, Yokohama, Kanagawa 230-0045, Japan

**Keywords:** Cell area, Chloroplast number per cell, Compensation, Leaf growth, Nuclear ploidy

## Abstract

**Background:**

Leaves are determinate organs; hence, precise control of cell proliferation and post-mitotic cell expansion is essential for their growth. A defect in cell proliferation often triggers enhanced post-mitotic cell expansion in leaves. This phenomenon is referred to as ‘compensation’. Several lines of evidence from studies on compensation have shown that cell proliferation and post-mitotic cell expansion are coordinately regulated during leaf development. Therefore, compensation has attracted much attention to the mechanisms for leaf growth. However, our understanding of compensation at the subcellular level remains limited because studies of compensation have focused mainly on cellular-level phenotypes. Proper leaf growth requires quantitative control of subcellular components in association with cellular-level changes. To gain insight into the subcellular aspect of compensation, we investigated the well-known relationship between cell area and chloroplast number per cell in compensation-exhibiting lines, and asked whether chloroplast proliferation is modulated in response to the induction of compensation.

**Results:**

We first established a convenient and reliable method for observation of chloroplasts *in situ*. Using this method, we analyzed *Arabidopsis thaliana* mutants *fugu5* and *angustifolia3* (*an3*), and a transgenic line *KIP-RELATED PROTEIN2* overexpressor (*KRP2* OE)*,* which are known to exhibit typical features of compensation. We here showed that chloroplast number per cell increased in the subepidermal palisade tissue of these lines. We analyzed tetraploidized wild type, *fugu5, an3* and *KRP2* OE, and found that cell area itself, but not nuclear ploidy, is a key parameter that determines the activity of chloroplast proliferation. In particular, in the case of *an3*, we uncovered that promotion of chloroplast proliferation depends on the enhanced post-mitotic cell expansion. The expression levels of chloroplast proliferation-related genes are similar to or lower than that in the wild type during this process.

**Conclusions:**

This study demonstrates that chloroplast proliferation is promoted in compensation-exhibiting lines. This promotion of chloroplast proliferation takes place in response to cell-area increase in post-mitotic phase in *an3*. The expression of chloroplast proliferation-related genes were not promoted in compensation-exhibiting lines including *an3*, arguing that an as-yet-unknown mechanism is responsible for modulation of chloroplast proliferation in these lines.

## Background

Leaf growth is driven by spatiotemporal control of cell proliferation and post-mitotic cell expansion. In the early developmental stage, cell proliferation actively occurs throughout leaf primordia. The proliferating region is maintained within a constant distance from the leaf base, then abruptly disappeared from distal to proximal regions of the leaf blade [1-5]. After arrest of mitotic cycling, cells show post-mitotic expansion with an intense increase in vacuolar volume. During leaf development, a defect in cell proliferation often triggers enhanced post-mitotic cell expansion. This phenomenon is termed ‘compensation’ [6-8] and is widely observed in seed plants including *Arabidopsis thaliana* (hereafter Arabidopsis) and *Oryza sativa*[9-11]. Interestingly, our recent study of compensation revealed that the two driving forces of leaf growth —proliferation and post-mitotic expansion of cells— are coordinately regulated during development [12]. Compensation is therefore considered to be a key phenomenon for understanding the control of leaf growth.

Enhanced post-mitotic cell expansion could be detected by observing leaf cells from a paradermal view and measuring their area. Kinetics of the cell-area changes indicated that increase in cell area takes place in three distinct manner in compensation-exhibiting lines represented by *angustifolia3* (*an3*) (enhanced rate of post-mitotic cell expansion), *fugu5* (prolonged post-mitotic cell expansion) and a cyclin dependent kinase inhibitor gene *KIP-RELATED PROTEIN2* overexpressor (*KRP2* OE) (increase in cell area in both mitotic and post-mitotic phases) [10,13]. The *an3* is well characterized among compensation-exhibiting lines. The *AN3* gene, also known as *GRF-INTERACTING FACTOR1*, encodes a transcriptional co-activator for leaf cell proliferation in Arabidopsis [14-16]. In the *an3* leaves, cell number is decreased by more than 70% but cell area is increased by 50% when compared with the WT [10,15-17]. For induction of enhanced post-mitotic cell expansion, a decrease in cell proliferation below a threshold is required [18]. This fact suggests that enhanced post-mitotic cell expansion in *an3* is not a simple result of a defect in cell proliferation, but a result of an active motion for leaf growth in response to a defect in cell proliferation. This idea is supported by our recent analysis of chimeric leaves for *AN3* expression: *an3* mutant cells are considered to actively produce and transmit an inter-cellular signal for enhanced post-mitotic cell expansion [12]. These studies have deepened our understanding of the mechanism of compensation, and hence leaf growth, at the cellular level.

On the other hand, subcellular aspects in compensation-exhibiting lines including *an3* have received less attention. Quantitative control of subcellular components is required for the proper functioning of leaf cells. Anatomical studies have revealed that the number of chloroplasts per cell is correlated with cell area [19-21]. In this study, we investigated the number of chloroplasts per cell in compensation-exhibiting lines to address whether subcellular aspect is affected in response to the induction of compensation.

Chloroplasts are derived from proplastids in meristematic cells, and multiply by division during leaf development. Two paralogous nucleus-encoded genes *PLASTID DIVISION1* (*PDV1*) and *PDV2* are involved in the chloroplast proliferation [22-24]. The expression for PDV1 and PDV2 in Arabidopsis occurs in the shoot apical meristem and in young leaf primordia, and then decreases in parallel with cessation of chloroplast proliferation [23,24]. Importantly, overexpression of *PDV1* and/or *PDV2* increases the number of chloroplasts, while a loss-of-function mutation in *PDV1* and/or *PDV2* has the opposite effect [22-24]. Other components involved in chloroplast proliferation have been also identified such as self-assembling cytoskeletal GTPase genes *Filamentous temperature sensitive Z1* (*FtsZ1*) and *FtsZ2*[25], a gene encoding J-domain containing protein *ACCUMULATION AND REPLICATION OF CHLOROPLASTS 6* (*ARC6*) [26], Min system-related genes [*MinC*, *MinD, MinE* and *MULTIPLE CHLOROPLAST DIVISION SITE 1* (*MCD1*)] [27,28]. Although involvement of these genes into chloroplast proliferation is apparent, it is known that overexpression or knockdown/knockout of them does not promote chloroplast proliferation [25-31]. Therefore, the extent of chloroplast proliferation depends primarily on the expression levels of *PDV*s. It is worth investigating the expression level of *PDV*s in compensation-exhibiting lines to determine whether chloroplast proliferation is modulated in response to the induction of compensation.

We here established a convenient method for counting chloroplasts in subepidermal palisade cells *in situ.* By use of this method, we investigate the relationship between cell area and chloroplast number per cell in compensation-exhibiting lines. Based on our results, we discuss the promotion of chloroplast proliferation in response to the enhanced post-mitotic cell expansion. In addition, we discuss whether the promotion of chloroplast proliferation occurs through an up-regulation of the expression levels of *PDV*s.

## Results and discussion

### Establishment of a method for counting chloroplasts *in situ*

**Figure 1:**
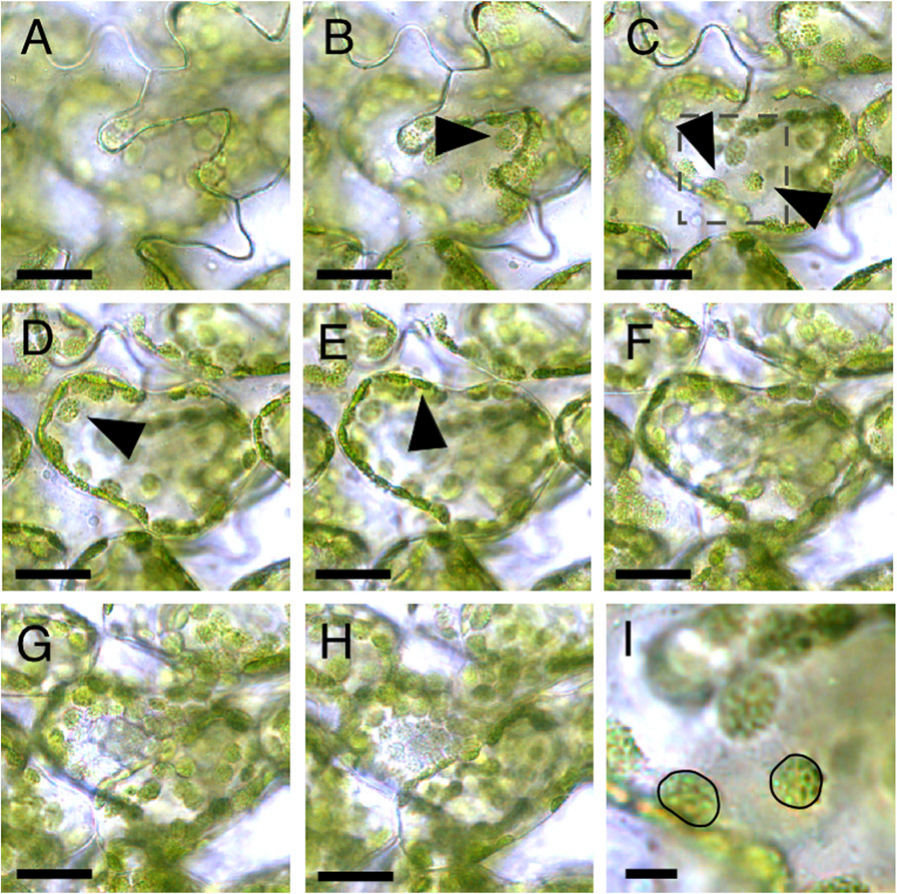
**Observation of chloroplasts within subepidermal palisade cells. (A-H)** Chloroplasts in subepidermal palisade cells observed using the method established here. Jigsaw-puzzle-shaped epidermal cells are seen in **(A)**. Focal depth is gradually deepened from **(A)** to **(H)**. Leaf samples are from 21-day-old plants. Arrowheads indicate focused chloroplasts. **(I)** Magnified view of the boxed region in **(C)**. Focused chloroplasts are outlined in black. Bars = 50 μm **(A-H)** and 10 μm **(I)**.

We first established a concise method for counting chloroplasts in the subepidermal palisade cells *in situ*. A reason for focusing on subepidermal tissue is that cellular phenotypes therein are well characterized in compensation-exhibiting lines [10,12,18]. To count chloroplasts, the first leaves from 21-day-old plants were immersed in 0.05% (v/v) Triton X-100 and 1% (v/v) glycerol under vacuum at room temperature, followed by observation. This method allowed us to clearly observe and count chloroplasts one by one by manually adjusting the focal plane up and down (Figure 1A-E and I). Cells below the subepidermal layer could also be observed (Figure 1F-H), ascertaining that this method is sufficient to obtain enough depth of focus to analyze the entire subepidermal layer *in situ*.

### Chloroplast number per cell increased in the leaves of compensation-exhibiting lines

**Figure 2:**
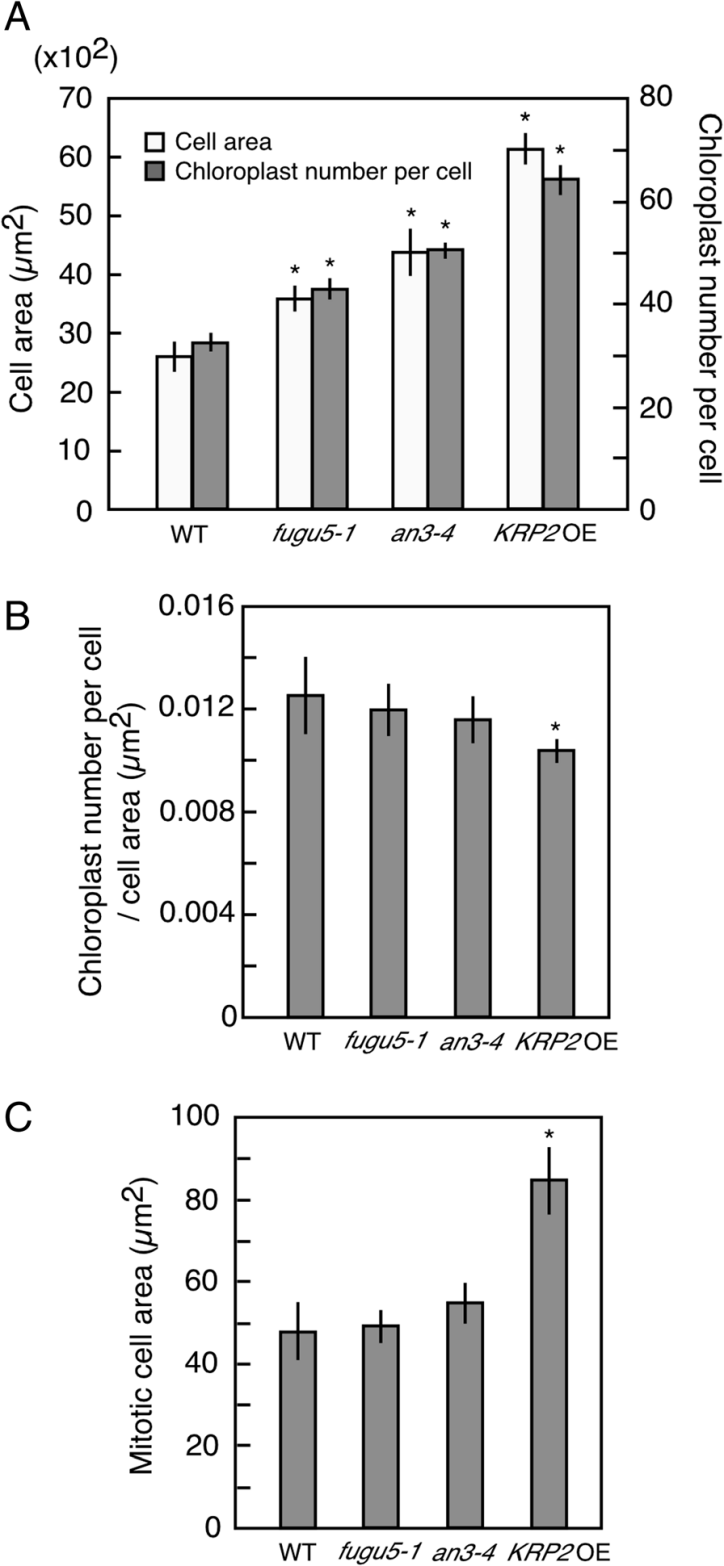
**Increased number of chloroplasts per cell in the leaves of compensation-exhibiting lines. (A)** Cell area and chloroplast number per cell measured in subepidermal palisade cells of WT, *fugu5-1*, *an3-4* and *KRP2* OE leaves. Leaf samples are from 21-day-old plants. The means ± SD of WT, *fugu5-1*, *an3-4* and *KRP2* OE lines are indicated. **(B)** Ratio of chloroplast number per cell/cell area in WT, *fugu5-1*, *an3-4* and *KRP2* OE leaves. **(C)** Area of mitotic subepidermal palisade cells of WT, *fugu5-1*, *an3-4* and *KRP2* OE leaf primordia. Leaf primordia are from 5-day-old seedlings. Asterisk indicates significant difference at P < 0.01 compared with WT (Student’s *t*-test).

We next investigated the number of chloroplasts per cell in the subepidermal palisade tissue of 21-day-old compensation-exhibiting lines including *fugu5*, *an3* and *KRP2* OE. Chloroplast numbers per cell increased by 30%, 67% and 141% in *fugu5-1*, *an3-4* and *KRP2* OE lines, respectively, when compared with the WT (Figure 2A). These data were reproducible with small standard deviations, confirming the validity of our method for counting chloroplast numbers per cell. Furthermore, this result indicates that one of the subcellular processes (*i*.*e*., chloroplast proliferation) is modulated in association with the induction of compensation irrespective of the manner by which compensation occurs. In addition, we observed leaf cells from a paradermal view and measured their area using the same leaf samples, which are used also for chloroplast counting. We found that cell area increased by 37%, 65% and 157% in *fugu5-1*, *an3-4* and *KRP2* OE, respectively, when compared to the WT (Figure 2A). These increased ratios of cell area are similar to those of the number of chloroplasts per cell: the value of chloroplast number per cell/cell area is constant to a similar extent in compensation-exhibiting lines comparable to the level of WT (although the value is slightly but significantly decreased in *KRP2* OE) (Figure 2B). These results suggest a tight relationship between final cell area and chloroplast number per cell in the subepidermal palisade tissue of compensation-exhibiting lines.

Chloroplast proliferation activity decreases with developmental progression in WT leaves [24]. On the other hand, our data indicate that the number of chloroplasts per cell is correlated with final cell area in the mature leaves, which is determined through the post-mitotic cell expansion. Mitotic cell area in 5-day-old *fugu5-1* and *an3-4* lines is similar to that in the WT (Figure 2C) [10], supporting our idea that chloroplast proliferation is modulated in response to the status of post-mitotic cell expansion in these mutants. We should carefully consider the contribution of post-mitotic cell expansion to promotion of chloroplast proliferation in *KRP2* OE, because area of mitotic cells in this line is about two fold larger than that in WT (Figure 2C) [10,13]. Therefore, chloroplast proliferation could be modulated also during mitotic phase of leaf cells. Establishment of method for precise enumeration of immature chloroplasts in mitotic cells would be helpful to further investigate this issue.

### Relationship between final cell area, nuclear ploidy and chloroplast number per cell

**Figure 3:**
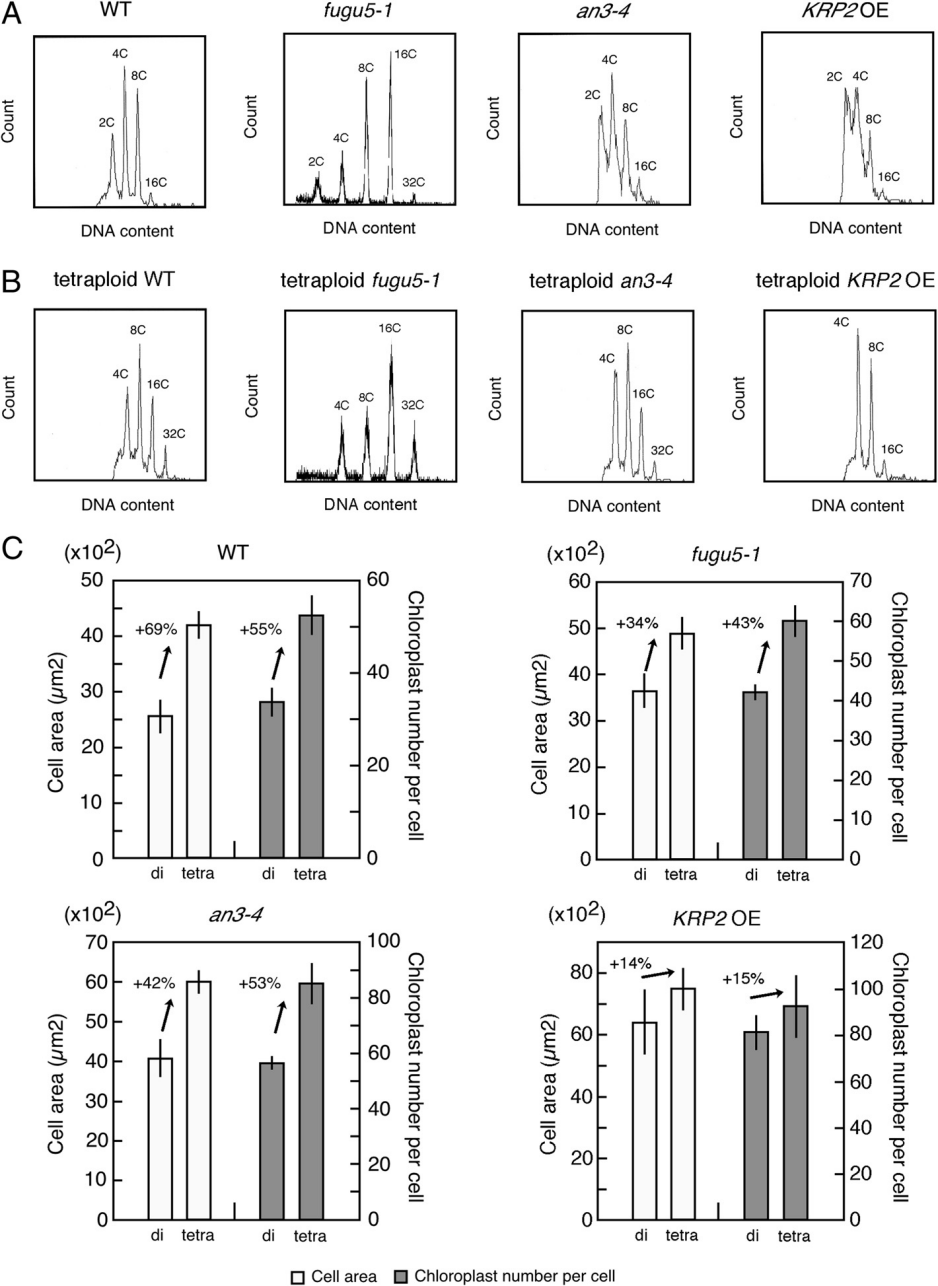
**Effect of nuclear ploidy on chloroplast proliferation in the leaves of compensation-exhibiting lines. (A and B)** Nuclear ploidy in diploid **(A)** and tetraploid **(B)** WT, *fugu5-1*, *an3-4* and *KRP2* OE lines analyzed through flowcytometoric analysis. Leaf samples are from 21-day-old plants. **(C)** Cell area and chloroplast number per cell measured in subepidermal palisade cells of WT, *fugu5-1*, *an3-4* and *KRP2* OE (diploid and tetraploid) leaves. di, diploid; tetra, tetraploid. Leaf samples are from 21-day-old plants. The means ± SD of WT, *fugu5-1*, *an3-4* and *KRP2* OE lines are indicated. Arrows indicate the difference between adjacent bars.

Final cell area often parallels the level of endoreduplication [32]. In addition, previous studies have shown that *AtCDT1a*, a component of the prereplication complex for DNA replication, is localized in both the nucleus and chloroplasts, and is involved in the regulation of endoreduplication and chloroplast division [33,34]. These facts suggest that nuclear ploidy is directly linked to chloroplast proliferation through the endoreduplication process. However, we concluded that nuclear ploidy does not directly linked to the chloroplast proliferation in compensation-exhibiting lines because the nuclear ploidy in these lines is varied, namely increased in *fugu5-1*, relatively normal in *an3-4* and decreased in *KRP2* OE compared to the WT (Figure 3A) [10,17,35].

Nuclear ploidy is increased through not only endoreduplication but also polyploidization. To further investigate the effect of increased nuclear ploidy on chloroplast proliferation, we established tetraploidized WT, *fugu5-1*, *an3-4* and *KRP2* OE lines (Figure 3B) and analyzed their leaves 21 days after sowing. We found that chloroplast number per cell increased by 55%, 43%, 53% and 15% in tetraploid WT, *fugu5-1*, *an3-4* and *KRP2* OE, respectively, when compared with diploid counterparts (Figure 3C). These rates of increase are different from that expected (100%) if chloroplast number per cell was linearly correlated with nuclear ploidy. Rather, these rates of increase are similar to those of cell area: in tetraploid WT, *fugu5-1*, *an3-4* and *KRP2* OE, cell areas increased by 69%, 34% 42% and 14%, respectively, compared to their diploid counterparts (Figure 3C). Together, we conclude that nuclear ploidy does not directly affect chloroplast proliferation, and final cell area is the key parameter for chloroplast proliferation.

### Enhanced post-mitotic cell expansion is required for promotion of chloroplast proliferation in *an3*

**Figure 4:**
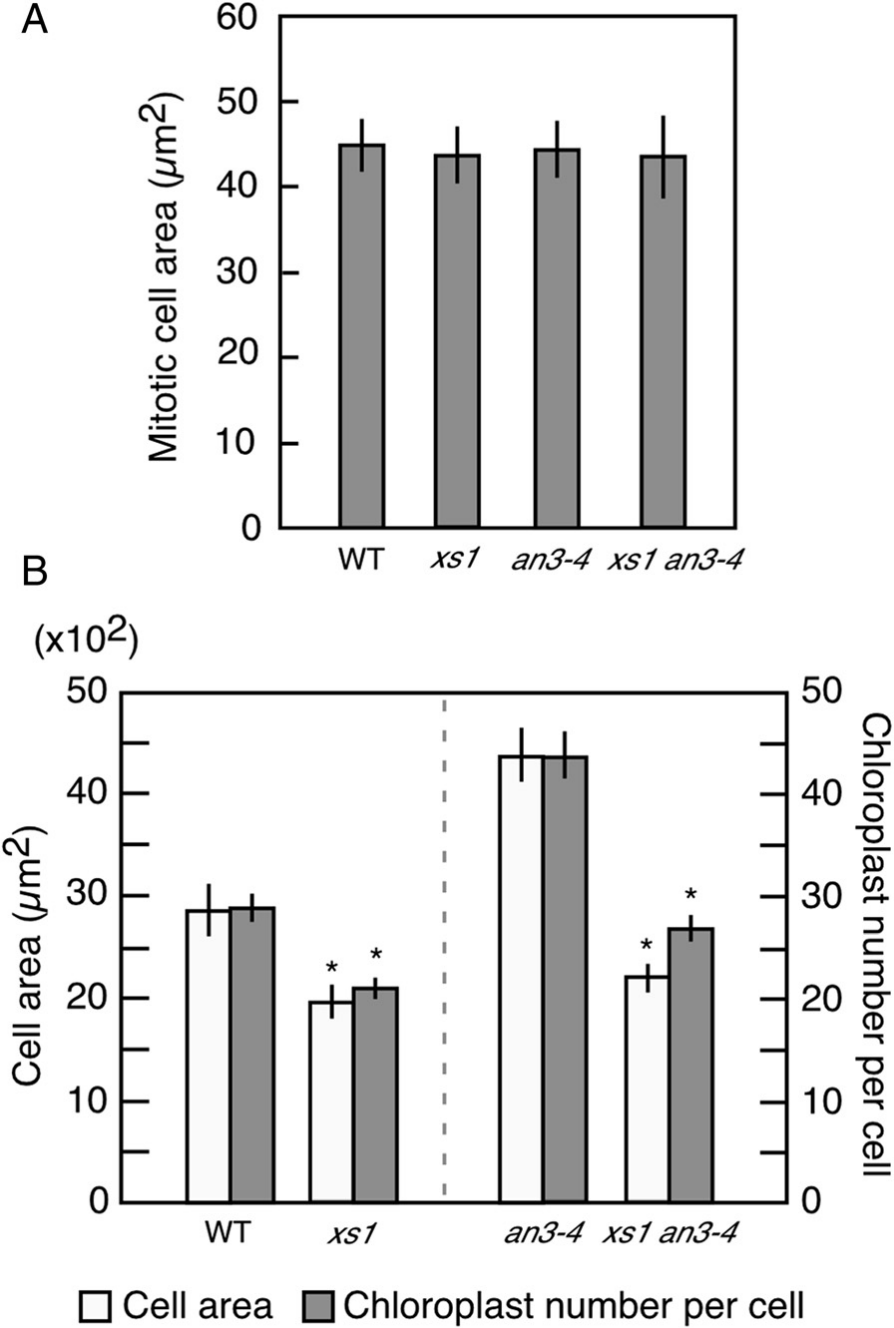
**Promotion of chloroplast proliferation in response to the enhanced post-mitotic cell expansion in *an3*. (A)** Area of mitotic subepidermal palisade cells of WT, *xs1*, *an3-4* and *xs1 an3-4* leaf primordia. Leaf primordia samples are from 5-day-old plants. The means ± SD from eight individual leaf primordia are indicated. **(B)** Cell area and chloroplast number per cell measured in subepidermal palisade cells of WT, *xs1*, *an3-4* and *xs1 an3-4* leaves. Leaf samples are from 21-day-old plants. The means ± SD of WT, *xs1*, *an3-4* and *xs1 an3-4* lines are indicated. Asterisk indicates significant difference at P < 0.01 between the two genotypes (WT versus *xs1*; *an3-4* versus *xs1 an3-4*) (Student’s *t*-test).

We previously identified *extra-small sister1* (*xs1*) mutant that shows decreased final cell area in leaves [17]. The *xs1* mutation suppresses enhanced post-mitotic cell expansion in *an3* genetic background [17], but does not affect cell area in mitotic phase irrespective of WT or *an3-4* genetic background (Figure 4A). We next investigate whether promotion of chloroplast proliferation depends on the cell-area increase in post-mitotic phase using *xs1 an3-4* double mutant line. The number of chloroplasts per cell decreased in subepidermal palisade tissue of 21-day-old *xs1 an3-4* when compared with *an3-4* in association with the decrease in final cell area (Figure 4B). We therefore concluded that chloroplast proliferation is promoted in response to the enhanced post-mitotic cell expansion in *an3-4*.

### Chloroplast proliferation is promoted in compensation-exhibiting lines without up-regulation of the expression of *PDV*s

It was previously believed that expression level of *PDVs* determines the rate of chloroplast proliferation because increased or decreased level of *PDVs* expression lead to an increase or decrease in chloroplast proliferation, respectively [23,24]. The expression for PDVs decreases along with leaf development in the WT, whereas our data indicate that chloroplast proliferation is promoted in response to increase in cell area. This fact suggests that the control of chloroplast proliferation might be more flexible than previously thought in response to the change in final cell area. If that is the case, whether the expression level of *PDV*s is up-regulated in response to cell-area change in leaves of compensation-exhibiting lines is an important question.

**Figure 5:**
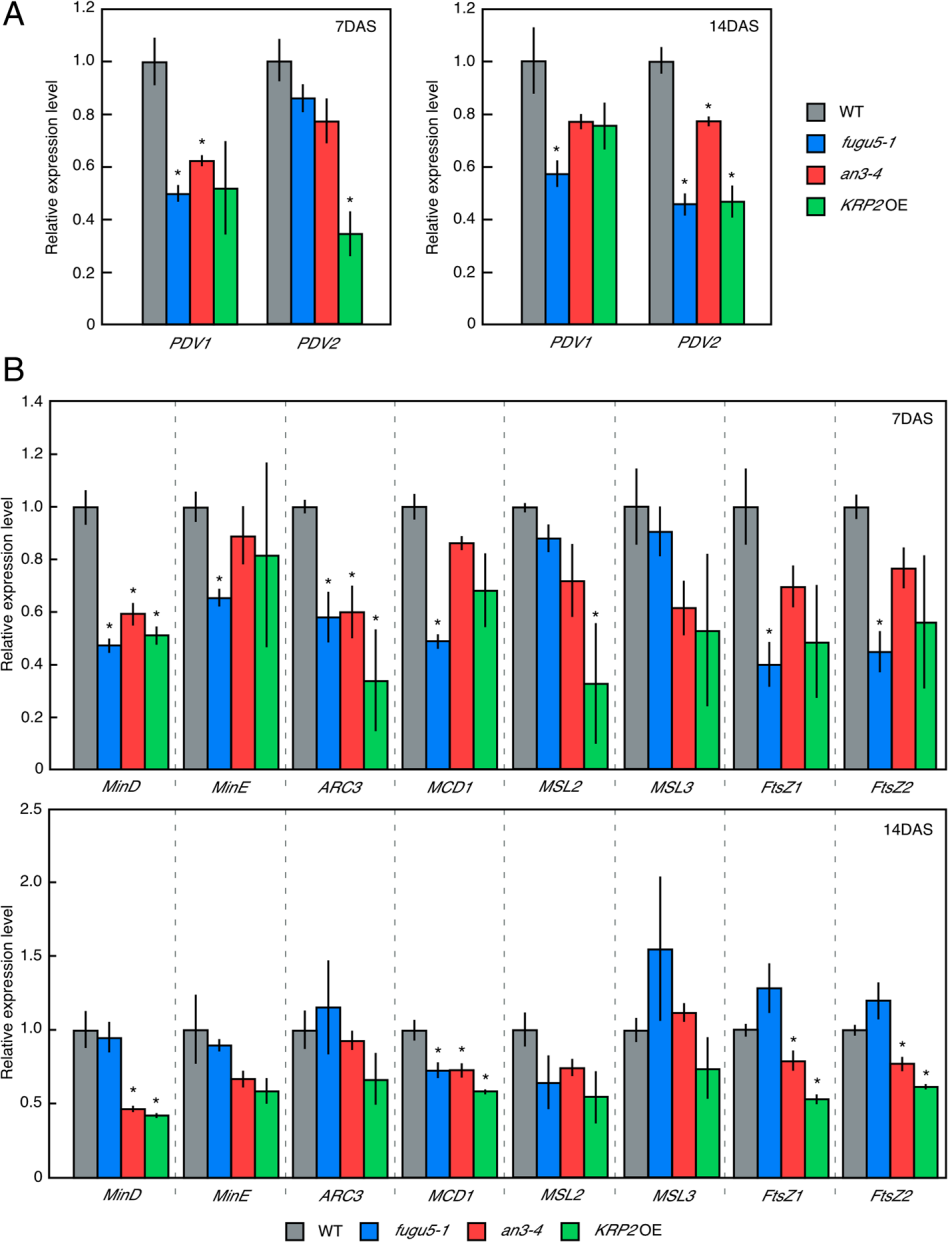
**Transcription levels of chloroplast proliferation-related genes in compensation-exhibiting lines. (A and B)** Expression levels of *PDV1* and *PDV2***(A)**, and other chloroplast proliferation-related genes **(B)** in WT, *fugu5-1*, *an3-4* and *KRP2* OE. Samples are from above-ground parts of 7-day-old seedlings and leaf primordia of 14-day-old seedlings. Transcription levels of chloroplast proliferation-related genes including *PDV1* and *PDV2* were normalized to that of *ACTIN2.* Data are means ± S.D. (n = 3, with triplicates in each sample). Asterisk indicates significant difference at P < 0.01 compared with WT (Student’s *t*-test).

To address this, we asked whether promotion of chloroplast proliferation occurs through the up-regulation of *PDV*s expression in compensation-exhibiting lines. We investigated the expression level of *PDV*s in the above-ground parts of 7-day-old seedling and leaf primordia in 14-day-old plants. Increase rate of cell area in *fugu5-1*, *an3-4* and *KRP2* OE is already higher than that in WT 14 days after sowing [10]. However, the expression level of *PDV*s in these compensation-exhibiting lines is similar to or lower than that in the WT (Figure 5A). This result indicates that the promotion of chloroplast proliferation in response to the increase in cell area in compensation-exhibiting lines does not depend on the up-regulation of *PDVs* expression. We further investigated the expression levels of other chloroplast proliferation-related genes in compensation-exhibiting lines, and found that their expression levels are also similar to or lower than that in WT (Figure 5B). This fact implies that promotion of chloroplast proliferation in compensation-exhibiting lines does not depend on the up-regulation of the expression of chloroplast proliferation-related genes.

## Conclusions

In this study we reported a reliable method for chloroplast enumeration *in situ* and demonstrated that chloroplast number per cell increases in compensation-exhibiting lines. Nuclear ploidy is not directly involved in the control of chloroplast proliferation in compensation-exhibiting lines. Of particular note is the finding that chloroplast proliferation is modulated during leaf development in response to the status of post-mitotic cells. In this process, the expression of *PDV*s and other chloroplast proliferation-related genes were not up-regulated, arguing an as-yet-unknown mechanism for promotion of chloroplast proliferation in response to cell-area change. These findings highlight a novel aspect of compensation, and, therefore, provide important insight into the fundamental understanding of leaf growth.

## Methods

### Plant materials and growth conditions

The WT accession of Arabidopsis used in this study was Columbia-0. Tetraploidization of WT, *fugu5-1*, *an3-4* and *KRP2* OE lines were carried out as described previously [36], followed by flowcytometory analysis to confirm their nuclear ploidy [37]. Plants were grown on rock wool at 22°C under 16 h light/8 h dark, and watered daily with 0.5 g L^-1^ Hyponex solution. Light at approximately 50 μmol m^-2^ s^-1^ was provided by white fluorescent lamps.

### Chloroplast enumeration

The first leaves from 21-day-old plants are infiltrated with 0.05% (v/v) Triton X-100 and 1% (v/v) glycerol, and then counted the number of chloroplasts (*n* = 160 cells from 8 leaves for each line). Detailed method is described in the Results and Discussion section.

### Measurement of cell area in leaves

After enumeration of chloroplast number per cell, the same leaf samples are subjected to analyze the cell area (*n* = 160 cells from 8 leaves for each line). Subepidermal palisade cells were observed under a light microscope (DMRX/E; Leica Microsystems). The leaf primordia was fixed in a formalin-acetic acid-alcohol (FAA) and cleared in a chloral hydrate solution (chloral hydrate, 200 g; glycerol 20 g; H_2_O, 50 ml) to measure the area of mitotic cells in the first leaves dissected from 5-day-old plants.

### Quantification of transcripts of chloroplast proliferation-related genes by qRT-PCR

Total RNA was extracted from above-ground parts of 7-day-old seedlings and primordia of the first leaves in 14-day-old plants using RNeasy Plant mini kit (QIAGEN) according to the manufacturer’s instructions. First-strand cDNA was synthesized from the extracted RNA using ReverTra Ace qPCR RT Master Mix with gDNA remover kit (TOYOBO). The PCR products were monitored by use of StepOnePlus real-time PCR system (Applied Biosystems) using Thunderbird SYBR qPCR mix (TOYOBO). Primers used were as follows: 5′ - CTTAACGCAATTCGAACCGC - 3′ and 5′ - TCCTGCTCTGTTCAAGCCG - 3′ for *PDV1*, 5′ - GCTGAACGGCTTTTGCGTAT - 3′ and 5′ - AATCAATCTCAGAGAGAGCCAGTTG - 3′ for *PDV2*, and 5′ - TCGGTGGTTCCATTCTTGCT - 3′ and 5′ - GCTTTTTAAGCCTTTGATCTTGAGAG - 3′ for *ACTIN2*, or as described elsewhere [38]. The expression levels of chloroplast proliferation-related genes were normalized by that of *ACTIN2* gene as an internal standard and expressed relative to the WT level (WT = 1). The specificity of PCR amplification was examined by monitoring the melting curves. Data were obtained from three independent biological replicates with triplicates in each sample, and were analyzed statistically.

## Authors’ contributions

KK and HT designed the research; GH helped design the research; KK performed the main parts of experiments; GH established *xs1 an3-4* double mutant line; NI and HT carried out tetraploidization of Arabidopsis lines; KK, GH, NI, MYH and HT wrote the paper. All authors read and approved the final manuscript.
